# Chikungunya Outbreak, South India, 2006

**DOI:** 10.3201/eid1410.070569

**Published:** 2008-10

**Authors:** Prabhdeep Kaur, Manickam Ponniah, Manoj V. Murhekar, Vidya Ramachandran, Ramakrishnan Ramachandran, Hari Kishan Raju, Vanamail Perumal, Akhilesh C. Mishra, Mohan D. Gupte

**Affiliations:** National Institute of Epidemiology, Chennai, India (P. Kaur, M. Ponniah, M.V. Murhekar, V. Ramachandran, R. Ramachandran, M.D. Gupte); Vector Control Research Center, Pondicherry, India (H.K. Raju, V. Perumal); National Institute of Virology, Pune, India (A.C. Mishra)

**Keywords:** Chikungunya, India, joint pain, outbreak, vector indices, dispatch

## Abstract

We investigated chikungunya outbreaks in South India and observed a high attack rate, particularly among adults and women. Transmission was facilitated by *Aedes aegypti* mosquitoes in peridomestic water containers, as indicated by a high Breteau index. We recommended vector control measures and health education to promote safe water storage practices.

Chikungunya fever (CHIK) outbreak was observed in India in December 2005. Phylogenetic analysis of the isolated virus showed a central-east African strain that was closely related to the strain from the Reunion Islands (*1*). Historically, the first outbreak of CHIK was reported in 1963 in Kolkata (*2*), and the last reported outbreak occurred in 1973 in Maharashtra (*3*). The reemergence of the virus may have been facilitated by human population migrations in the Indian Ocean region (*4*). Since December 2005, cases of CHIK were reported from several Indian states including Andhra Pradesh, Maharashtra, Karnataka, Tamil Nadu, and Madhya Pradesh. We investigated CHIK outbreaks in Andhra Pradesh and Tamil Nadu to describe the outbreak, estimate the incidence of subclinical infection, and propose control measures.

## The Study

In Andhra Pradesh, on the basis of the reports obtained from the local primary health centers, we selected Mallela village (2006 population: 1,965) of Kadapa district. In Tamil Nadu, we investigated the outbreak in Gowripet area (2006 population: 2,649) of Avadi, a suburban locality of Chennai City where a large number of persons with fever and joint pain were reported in June 2006.

In both settings, we conducted a door-to-door search of all households for case-patients who had acute onset of febrile illness and joint pain. We described the outbreak in terms of time, place, and person. We collected blood samples from case-patients after obtaining informed consent. We also collected blood samples from consenting asymptomatic persons in Mallela village to assess the incidence of subclinical infections. We tested serum samples for immunoglobulin (Ig) M antibodies against CHIK virus using IgM-capture ELISA at the National Institute of Virology, Pune (*1*).

For the larval survey in Mallela, we selected a representative sample of households after stratifying the village by attack rates; in Avadi, we surveyed all households. We calculated house index (HI), the proportion of houses having containers with larvae, and the Breteau index (BI), the number of containers positive for mosquito larvae per 100 houses. In Avadi, we mapped all the case households using a geographic positioning system device (ArcGIS version 8.02; ESRI, Redlands, CA, USA). We divided the area into 200-m^2^ grids to determine correlation between BI and attack rates.

We identified 242 case-patients meeting the case definition in Mallela (attack rate: 12%; no deaths) and 575 case-patients in Avadi (attack rate 22%; no deaths). The median number of joints affected in Mallela and Avadi was 3 and 4, respectively. The most common joints involved were ankle, knee, wrist, and small joints of hands in both settings. In addition to fever and joint pain, 59 (10%) and 28 (12%) case-patients in Mallela and Avadi, respectively, reported a rash. Case-patients were bed-ridden for an average of 6 to 7 days in both settings.

**Figure 1:**
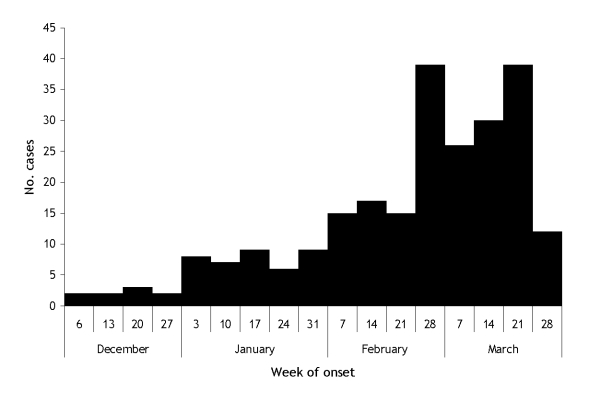
Chikungunya cases by week of the onset, Mallela village, Kadapa district, Andhra Pradesh, India, 2005–2006.

Attack rates were higher among persons >15 years of age and females in both settings (Table). In Mallela, cases began occurring during December 2005 and peaked during the first week of March 2006 (Figure 1). In Avadi, cases began occurring during May 2005 and peaked during the third week of June 2006 before declining. In Mallela, attack rates in different areas of the village ranged between 0% and 21%. Neighborhoods where persons of lower socioeconomic status resided in households with a single room and no water storage facility had the lowest attack rates. In contrast, neighborhoods where people lived in pucca houses (houses made with brick and mortar) with plenty of water storage containers had higher attack rates. Both outbreaks occurred during the summer months with temperatures ranging from 30°C to 44°C in Mallela and 27°C and 37°C in Avadi.

**Table:** Characteristics of chikungunya outbreak in South India, 2005–2006*

Area	Mallela, Andhra Pradesh	Gowripet, Avadi, Tamil Nadu
Setting	Rural	Urban
Population	1,965	2,649
Attack rate		
Overall	12% (n = 242)	23% (n = 575)
Age, y		5.8%
0–4	2.4%	20%
5–14	3.6%	24%
15–44	12.9%	22.8%
>45	22.2%	
Gender		18.9%
M	9.9%	
F	14.8%	24.5%
Chikungunya-specific IgM positivity	67% (90/134)	56% (5/9)
Subclinical infection		
No. tested	100 in all age groups	ND
Age-specific prevalence of IgM antibodies		
5–14 y	4%	ND
15–44 y	8%	ND
>45 y	3%	ND

*Ig, immunoglobulin; ND, not done.

**Figure 2:**
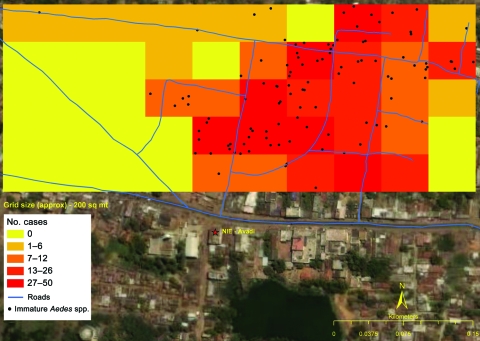
Chikungunya cases and presence of *Aedes* spp. immature mosquitoes, Gowripet, Avadi, Chennai, Tamil Nadu, India, 2006.

We conducted a larval survey in 56 houses in Mallela and all 657 households in Avadi. In both settings, water was scarce and residents used a variety of water storage containers, including plastic/earthen pots, plastic drums, and cement cisterns. The mean number of containers per household was 9 in Avadi. The HI and BI were 30% (17/56) and 39% (22/56), respectively, in Mallela. The HI and BI were 23% (148/657) and 35% (228/ 657), respectively, in Avadi. We observed a weak but significant correlation between attack rates by 200-m^2^ grids and BI (r = 0.37, p = 0.04) (Figure 2).

In Avadi, 5 of the 9 blood samples collected from the case-patients were positive for IgM antibodies. In Mallela, 90 (67%) of the 134 blood samples collected from case-patients were positive for IgM antibodies. In Mallela, we included 100 asymptomatic volunteers for serologic testing (median age 25 years, range 6–70); 33 were <15 years of age and 51 were female. Fifteen of the 100 asymptomatic persons had detectable IgM antibodies against CHIK virus. Our findings suggest that the apparent to inapparent case ratio was almost 1:1. There was no significant difference in the prevalence of IgM antibodies between age groups >15 years of age and <15 years of age (16% vs. 12%; p = 0.40) or between sexes (16% in males vs. 14% in females, p = 0.47).

## Conclusions

The key finding of our Chikungunya outbreak investigation was a high attack rate, particularly among adults and females. The outbreak occurred nearly 32 years after the last reported outbreak of CHIK in 1973 and was characterized by a prolonged duration. Transmission was facilitated by larvae of *Aedes aegypti* mosquitoes in peridomestic water containers. Our findings also suggest a considerable number of subclinical infections during the outbreak.

The explosive nature of the outbreak with high attack rates might be due to the absence of herd immunity to the central/East African genotype of the CHIK virus isolated in India and other countries in Indian Ocean region. CHIK outbreaks reported in the 1960s and 1970s from India were related to the Asian genotype of the virus (*1*). Unlike with dengue fever, we observed higher attack rates among adults than among children. We observed higher attack rates in females as observed in other countries (*5**–**7*).

The epidemic curves observed in both study sites indicated that transmission was ongoing for a considerably long period. The short flight range of the vector likely resulted in gradual transmission of infection among hosts in both communities.

The BI threshold for predicting CHIK transmission in India is not available. However, in both areas, BI was higher than 5 (35%–39%), which is the threshold for dengue transmission per the guidelines of the National Institute of Communicable diseases, India (*8*). The weak positive correlation between attack rate and BI in Avadi could be due to high vector density, which might distort the association between vector indices and clinical CHIK.

The limitations of our study were the lack of uniform methods for investigating 2 outbreaks and the convenient sampling method used to estimate incidence of subclinical infection. CHIK outbreaks occurred during the peak summer season because of favorable environment for *Ae*. *aegypti* mosquitoes in the form of water storage containers that were used in the absence of regular water supply and acute water scarcity. Most of these containers were either uncovered or partially covered and were not cleaned at regular intervals. Thus, a regular water supply that negates the need for water storage, education of the public for safe water storage measures, and environmental control are much needed public health measures to combat future CHIK outbreaks.
